# Retrospective analysis of radial EBUS outcome for the diagnosis of peripheral pulmonary lesion: sensitivity and complications

**DOI:** 10.3402/ecrj.v2.28947

**Published:** 2015-12-17

**Authors:** Amal Durakovic, Henrik Andersen, Anders Christiansen, Irena Hammen

**Affiliations:** Department of Pulmonary Medicine, Gentofte University Hospital, Hellerup, Denmark

**Keywords:** cancer, endoscopic intervention, radial EBUS

## Abstract

**Background:**

The purpose of the current study was to clarify the sensitivity and complication rate of the radial (endobronchial ultrasound, EBUS) without the use of guide-sheath (GS) and fluoroscopy for lung cancer (LC), by measuring the distance from the orifice of the bronchus to the pulmonary lesion, as well as to analyze factors that can predict the diagnostic outcome.

**Materials and methods:**

A total of 147 patients with peripheral pulmonary lesions (PPL) underwent radial EBUS-guided transbronchial biopsy (TBB) in between August 1, 2013, and August 31, 2014. We analyzed retrospectively radiological data, diagnostic work-up in everyday clinical settings, final diagnosis and complication rates, as well as factors influencing the diagnostic outcome.

**Results:**

Around 63.9% of PPLs were visualized by ultrasound. A definitive malignant diagnosis was established in 39 patients (26.5%) using radial EBUS. In the remaining 108 patients, additional procedures were performed. We missed LC diagnosis in 40 cases that results in a sensitivity of 49%. For malignant lesions visualized by radial EBUS, the sensitivity was 60%, compared with 24% for not visualized lesions. For malignant lesions, logistic regression was performed to identify the factors that had significant influence on visualization of the lesion and on diagnostic yield. Logistic regression analysis showed significant odds ratios (OR) for visualization depending on location of the lesion; upper lobe lesions were identified more frequent with OR of 3.85 (95% CI 1.42 – 10.98, *p*=0.009). Size above 30 mm had a non-significant OR of 2.11 (95% CI 0.80−5.73, *p*=0.134) for visualization.

Diagnostic yield was only significantly influenced by visualization with the radial EBUS, OR 3.70 (95% CI 1.35−11.02, *p*=0.014). Location (*p*=0.745) and size above 30 mm (*p*=0.308) showed no significant increase in diagnostic yield.

Other lesion characteristics defined on computed tomography, such as distance to carina and pleura, did not show any significant influence on the diagnostic yield. The complications rate was low with three cases of pneumothorax.

**Conclusion:**

Radial EBUS has definitely its place in the diagnostic work-up of PPL, especially for the lesions that can be visualized by radial ultrasound. However, prospective randomized controlled studies are necessary to raise the diagnostic yield and to define factors that can predict the outcome, which will consequently enable selection of the ‘right’ patients for this diagnostic procedure.

Lung cancer (LC) is the most common cancer worldwide, both in terms of incidence and mortality. Different screening programs, using chest radiographs and computed tomography (CT), have been tried, but in many cases these lead to the presentation of peripheral pulmonary lesions (PPL) of unclear significance. Pulmonologists are confronted with the choice of the optimal diagnostic strategy. Especially in the case of a positive fluorine-18 fluorodeoxyglucose (F-FDG) PET/CT result, which is always suspected to present a malignancy, diagnostic work-up is often complex and includes several interventional procedures to obtain pathological samples. The ability to confirm a pathologic diagnosis heavily relies on obtaining adequate cellular material. CT-guided percutaneous transthoracic needle biopsy of pulmonary lesions is one of the well-established procedures for the diagnosis of PPL, but not every lesion can be reached and the risk of pneumothorax is high, reported to be between 15 and 43% in different studies (1).

Flexible bronchoscopy has visual limitation up to sub-segmental bronchial level, and so in case of a peripheral lesion, it can only offer the possibility of blind transbronchial biopsy (TBB). Fluoroscopy has been used for many years to increase the sensitivity of TBBs and to reduce the risk of pneumothorax. The diagnostic yield ranges between 48 and 80% for malignant and 35 to 50% for benign PPLs (2). Diagnostic yield depends on the size and localization of the lesion; for the lesions less than 2 cm the diagnostic yield can be as low as 40% (3).

Several guided-bronchoscopy methods have been developed to increase the diagnostic yield of TBBs for pulmonary lesions; such as virtual bronchoscopic navigation, electromagnetic navigational bronchoscopy (EMN), and radial endobronchial ultrasound (EBUS). The last one is an ultrasound modality with a 20 MHz transducer inside a guide-sheath (GS); inserted through the working channel of the bronchoscope the GS provides an ultrasound image of the PPL and the bronchial wall. Biopsy instruments can be inserted through the GS, so samples can be sequentially obtained by keeping the GS in the lesion.

The purpose of the current study was to clarify the sensitivity and complication rate of the radial EBUS without the use of GS by measuring the distance from the orifice of the bronchus to the pulmonary lesion.

## Materials and methods

### Patients

We performed a retrospective review of the medical records of the 1,100 consecutive patients who were referred to our multidisciplinary Centre of Thoracic Oncology and Pulmonology at Odense University Hospital, Denmark, between August 1, 2013, and August 31, 2014, for evaluation of presumed intrathoracic malignancy. In this period, 147 patients with PPL underwent bronchoscopy with EBUS guidance, using a radial scanning ultrasound probe in peripheral airways, as the first choice of diagnostic intervention. The patients were chosen according to the practical consideration, when we thought that other procedures, for example, CT-guided biopsy or bronchoscopy would either be non-diagnostic or would have a high risk of pneumothorax.

We analyzed retrospectively our diagnostic work-up in this group and the following data were recorded: radiological information (size of the peripheral lesion and lobar position, distance to carina and pleura), pathological results, final diagnosis, and complications.

Because this study was a retrospective analysis, we did not submit any documents to the internal review board at our institution.

### CT/PET-scan

A PET/CT scan was performed in all cases. We analyzed scans for the maximal size of the solid component of the lesion and lobar position. A PPL was defined as a solitary pulmonary lesion that was surrounded by pulmonary parenchyma and was not endoscopically visible by bronchoscopy.

### Radial EBUS without GS

Bronchoscopy was performed with 2% topical lidocaine and intravenous sedation with midazolam and fentanyl. We used Olympus BF-1T180 bronchoscopes.

Radial EBUS was performed with an endoscopic ultrasound system Olympus EU ME 30; ultrasound probes 20 MHz Olympus UM-S20-17S and MAJ-935 probe driving unit. Procedures were performed by five operators, all with more than 4 years of experience in bronchoscopy. Radial scanning probe was inserted in the airways via flexible bronchoscope. All visible, relevant segments were scanned using ultrasound. If the lesion was visualized, the distance between the bronchoscope inserted in the orifice of the bronchus and the lesions was measured. The EBUS probe was then removed and forceps were introduced through the bronchoscope channel. Biopsies (at least four forceps biopsies) were performed in the same subsegment and at the same distance from the orifice of the bronchus. If the lesion was not visualized, blind forceps, brush, and bronchial wash biopsies were obtained from the relevant lung segments without fluoroscopy.

### Statistical analysis

Continuous variables are expressed as mean±standard deviation. Sensitivity was calculated according to standard definitions. The primary method of analysis to determine factors that influenced diagnostic outcome of radial EBUS was multiple logistic regression analysis. We looked primarily at the type of disease (cancer diagnosis), lesion size, lobar localization, distance from carina and pleura, as well as visualization of the lesion with radial EBUS. Two different size categories were chosen (Median size of tumor was 28 mm; 30 mm was selected as a cut off for large and small infiltrates). Statistical significance was established at the *p*<0.05 level. Logistic regression was performed using R version 3.2.2. For non-parametric data, a Wilcoxon test was performed. Non-parametric data were described as median, minimum, and maximum values. Chi-square test was performed where indicated.

## Results

We examined 147 patients (67 men and 80 women) with PPL using TBB guided by radial EBUS without GS. The median age was 67.8±11.3 years (range 24–89 years). The size of the lesion was recorded by its longest diameter on the PET/CT scan. The diameter of the lesions was wide ranged: from 7 mm to 90 mm. Most of the lesions were between 21 and 30 mm (29%), followed by small lesions (under 20 mm) (20%). The mean (±SD) diameter of the PPLs was 28±18.0 mm. The majority of the PPLs were localized in the upper lobes (32% in the right upper lobe and 27% in the left upper lobe). The PET/CT scan appearance of the PPLs showed FDG-uptake in all cases.

**Fig. 1:**
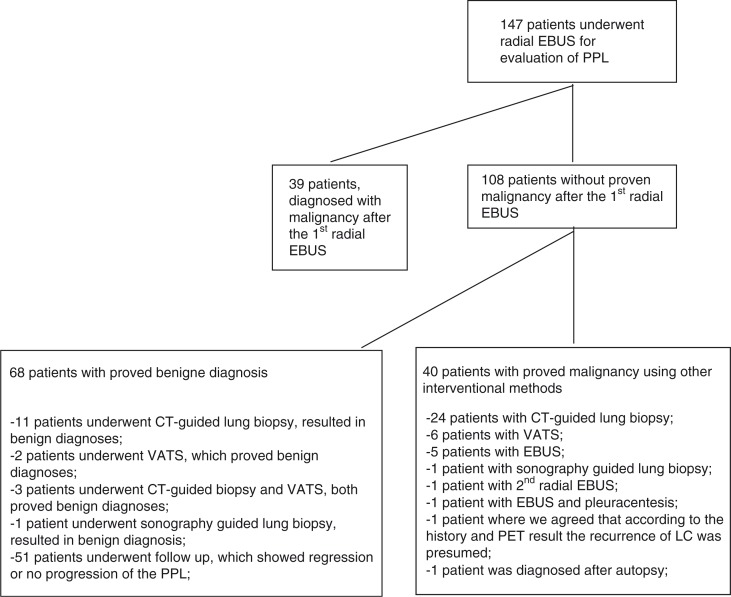
Flow diagram illustrating diagnostic work-up of all the patients with PPLs.

Of the 147 PPLs, 94 (63.9%) were visualized by ultrasound using radial EBUS. In 94 patients, we performed forceps biopsies (at least four biopsies) to obtain histological tissue samples: in 69 cases brush biopsies and in 114 cases bronchoalveolar wash samples were taken. Malignant diagnosis was made in 39 cases (26.5%) using radial EBUS. Table 1 shows the distribution of malignant pathologic diagnoses, and Fig. 1 shows a flow diagram illustrating diagnostic work-up of all the patients.

**Table 1:** Malignant diagnoses obtained by radial EBUS

Malignant	
- Adenocarcinoma	18
- Squamous cell carcinoma	7
- Small-cell carcinoma	1
- Undifferentiated NSCLC	6
- Neuroendocrine	1
- Metastasis	3
- Mamma carcinoma	1
- Renal cell carcinoma	1
- Malignant melanoma	1
Total	39

A definite diagnosis was considered established when histological or cytological results were defined as malignant disease. In other cases, for example, when pathology showed inflammatory change or nothing pathologic was found in the lung tissue, biopsy was designated as non-diagnostic. Consequently, we continued our diagnostic work-up by proceeding to the other interventional techniques (CT-guided transthoracic lung biopsy and VATS) or followed up with PET/CT scan, when the risk of malignancy was low, compared with the high risk of complications of the other interventional procedures.

In 39 cases, pathological tests (forceps biopsies) proved malignancy (35 cases of LC and four cases of metastases from other cancers). Thus, we missed cancer diagnosis with radial EBUS without GS in 40 cases that results in a sensitivity of 49% for LC.

For malignant lesions visualized by radial EBUS, the sensitivity was 60% (32 out of 52), compared with only 24% for not visualized (7 out of 24).

For malignant lesions, logistic regression was performed to identify the factors that had significant influence on visualization of the lesion and on diagnostic yield. Logistic regression analysis showed significant odds ratios (OR) for visualization depending on location of the lesion; upper lobe lesions were identified to be more frequent with OR of 3.85 (95% CI 1.42−10.98, *p*=0.009). Size above 30 mm had a non-significant OR of 2.11 (95% CI 0.80−5.73, *p*=0.134) for visualization (Table 3).

Diagnostic yield was only significantly influenced by visualization with the radial EBUS, OR 3.70 (95% CI 1.35−11.02, *p*=0.014). Location (*p*=0.745) and size above 30 mm (*p*=0.308) showed no significant increase in diagnostic yield (Table 2).

**Table 2:** Statistical tests, comparing characteristics of malignant lesions where diagnosis was provided by radial EBUS with non-diagnostic procedures

Variable	Radial EBUS (+)	Radial EBUS (−)	Statistics
Lesion size	40 (13–90)	31.5 (14–70)	*p*=0.075*
Distance PPL-pleura	15 (0–68)	12 (0–70)	*p*=0.415*
Distance PPL-carina	63.5 (10–127)	61 (8–135)	*p*=0.658*
Upper lobe	24	22	*p*=0.745**
Lower lobe	15	16	

Non-parametric statistical tests used for lesion size, distance to pleura, and carina.

* Wilcoxon's test.

** Chi-square test.

We also compared PET-/CT-related characteristics of malignant lesions: those where we proved diagnosis with radial EBUS with those where diagnosis was ensured by other methods (Table 2). No significant difference was identified for distance to carina or pleura. There was no significant difference in visualization depending on distance to either pleura or carina (Table 3).

**Table 3:** Statistical tests, comparing characteristics of visualized malignant lesions versus non-visualized

Variable	Visualization (+)	Visualization (−)	Statistics
Lesion size	39 (13–90)	30 (14–85)	*p*=0.094*
Distance PPL-pleura	19 (0–70)	(0–64)	*p*=0.541*
Distance PPL-carina	(8–127)	(10–135)	*p*=0.771*
Upper lobe	37	16	*p*=0.007**
Lower lobe	9	15	

* Wilcoxon's test.

** Chi-square test.

In addition, we evaluated whether our samples from TBB obtained with radial EBUS delivered enough material for the genotyping of EGFR. In our study 18 PPLs were adenocarcinomas; in eight cases there was enough material to conduct EGFR analysis and in 10 cases we unfortunately did not obtain enough material.

The complication rate was low with three cases of pneumothorax (in two cases requiring chest tube). There were no cases of lung bleeding or any other complications.

## Discussion

In the current study, 63.9% of the lesions were visualized with ultrasound using radial EBUS.

The sensitivity for cancer was 49%, rising to 60% for the visualized malignant lesions.

Several groups have evaluated the effectiveness of TBB using EBUS-GS for diagnosing of PPLs. The diagnostic yield has fluctuated from 53% up to 77% (2, 4, 5). The great variability in results is likely because of the difference in experience of the operators, in the use of different techniques and biopsy methods.

Chung et al. evaluated the effectiveness of EBUS-guided TBB using distance measurement from the orifice of the bronchus to the detected lesion as an alternative method to EBUS-GS and fluoroscopy (6). They reported a total diagnostic yield of 78.9% for PPLs with the size from 10 mm to 44 mm. Fuso et al. used the same method in a retrospective study of 662 patients with PPLs with a size of 36±20 mm; 75% of the lesions were visualized. EBUS-guided TBB had a total sensitivity of 71% for the diagnosis of LC and for the diagnostic accuracy of 77% (4). Rivera et al. (7) performed a systematic search of the MEDLINE, Healthstar, and Cochrane Library databases covering studies up to 2011 comparing the outcome of different bronchoscopic methods in the patients with presumed LC. The sensitivity of radial EBUS was 34% for the lesions up to 20 mm and 63% for the bigger lesions.

Therefore, the sensitivity of 49% in our study is comparable with the results of other centers. It has to be considered that 29% of the lesions were between 21 and 30 mm, and 20% were under 20 mm (20%), which could explain that sensitivity was on the lower end comparing with the other studies.

Different groups have evaluated factors influencing the yield of the EBUS-guided TBB. The most obvious and therefore the most often analyzed factor is the size of the peripheral lesion. A study by Yamada et al. looked at the factors influencing the yield of the EBUS-guided TBB in a cohort of 155 patients with PPL (8). The efficacy was 67% and was significantly lower for the lesions smaller than 15 mm (40%) than for the lesions with the diameter of 15–30 mm (76%) (9). Yoshikawa et al. (2) analyzed the efficacy of EBUS-GS in a cohort of 123 patients with PPLs, the efficacy for lesions >20 mm in diameter (75.6%) was significantly higher than for smaller lesions (29.7%).

Several groups have also looked at other factors that can possibly predict the diagnostic outcome of TTB guided with radial EBUS. Some studies reported that difference in visualization resulted in higher diagnostic yield between malignant and benign lesions. For example, Tay et al. reported that malignant lesions had a higher visualization rate (85%) than benign lesions (66%) in the cohort of 196 patients with PPL (8). Tamiya et al. also showed approximately the same correlation in the study of 68 patients (83.7 and 68.0% for the malignant and benign lesions, respectively) (10).

The same study also reported a significant difference in the efficacy between cases, when the EBUS probe was localized within versus adjacent to the lesion 92.1% versus 60.0%, respectively. Also, in the report from Yamada et al., lesions in which the probe was positioned within the PPL had a higher diagnostic yield (83%) than PPLs in which the probe was positioned adjacent to the PPL (61%) or outside the PPL (4%) (9). In the study by Huang et al., the location of the PPL on CT scans and position of the probe were independent predictors of the diagnostic yield by EBUS-guided TBB as well (11).

In the current study, the diagnostic yield for malignant lesions in the multivariate analysis was only significantly influenced by visualization with the radial EBUS, OR 3.70 (95% CI 1.35−11.02, *p*=0.014). Location (*p*=0.745) and size above 30 mm (*p*=0.458) as well as distance to carina and pleura did not show any significant increase in diagnostic yield.

Our study has several limitations. First, it was a retrospective chart review in a single institute and evidence level is lower than that of prospective studies. Second, the procedures were performed without the use of GS or X-ray fluoroscopy by different operators. Hypothetically, it could be assumed that if the procedure had been performed with the GS and fluoroscopy by only one operator, the diagnostic yield would have been higher. In addition, this method has been introduced in our clinic not so long ago, so it could be assumed that there is also a learning curve. It could be interesting to register diagnostic yield for each operator over a period, but that would be challenging in the everyday clinical praxis.

It is also important to choose the ‘right’ patients for this procedure. Because the method is relatively new, we are still learning how to select the patients. Compared with the other diagnostic methods of PPLs, for example, CT-guided lung biopsy, radial EBUS has two major advantages: low complications rate, especially low pneumothorax risk, and we can reach lesions that are located near to the bronchial tree and far from the pleura. Alternative diagnostic methods, such as CT-guided biopsy, would be connected to a much bigger risk of pneumothorax in particular because of the central location of the lesions. In some cases, the only alternative diagnostic methods would be VATS or even lobectomy which are definitely more complicated and risky procedures.

An important learning point for us was that the diagnostic yield was clearly dependent on the visualization of the lesion by ultrasound using the radial EBUS. In addition, the visualization was influenced by location; the lesions in the upper lobes were visualized more frequently. Unfortunately, we were not able to identify any other factors, which could predict the success of the procedure – size, location, or distance to carina or pleura did not have any significant influence on the diagnostic yield.

Recently, other alternative methods for the diagnostic of PPL, such as EMN bronchoscopy and virtual bronchoscopy (VB), have been developed. For example, the study group of Steinfort et al. examined 236 patients with the lesion size 22.8±12.4 mm (12). PPLs were visualized using EBUS +VB alone in 77% and were diagnostic in 71.3% of these. The additional use of EMN improved overall visualization yield to 85% and overall diagnostic yield to 58.4%. Sensitivity for diagnosis of LC was 70%.

In conclusion, PPL, especially those under 20 mm, stays a diagnostic challenge. That is why the work-up should include all available methods to reach the best possible diagnostic yield. The contribution of different methods still remains uncertain and needs further development. The radial EBUS has definitely its place in the diagnostic work-up of PPL's. However, the prospective randomized controlled studies in the future are necessary to raise the diagnostic yield, as well as further evaluation of factors that can predict the outcome, enabling adequate patient selection for this procedure.

Based on our results and experience, the probable algorithm for the diagnostic work-up of the PPL algorithm could be:

1. Identifying PPLs localized near pleura where we can expect to get diagnosis from CT-guided lung biopsy without a high risk of pneumothorax.
2. PPLs localized further in the lung parenchyma could undergo radial EBUS evt. combined with fluoroscopy and/or VB.
3. If the procedure is not diagnostic and EMN is available, this method should be used to rise the diagnostic yield.
4. However, in some cases, only surgical procedures, for example, VATS can provide diagnosis, and this option should be discussed in the multidisciplinary team.
